# Supplementary material for: Modelling digital and manual contact tracing for COVID-19. Are low uptakes and missed contacts deal-breakers?
Source: PLoS One. 2021 Nov 18;16(11):e0259969. doi: 10.1371/journal.pone.0259969 (PMC8601513; doi:10.1371/journal.pone.0259969)
Supplement: S1 File — Contains a link to the repository that maintains our open-source model, further discussions on other epidemic statistics we captured, and more charts illustrating the effects induced by varying the contact tracing parameters. (PDF) [file pone.0259969.s001.pdf]

Supporting information

Open-source model and data

The open-source implementation of our model can be consulted at:
`https://github.com/andrei-rusu/contact-tracing-model`.

The statistics our simulations captured can be analyzed in full by following:
`https://doi.org/10.6084/m9.figshare.14101946`.

Simulation statistics

Aside from the metrics analyzed in the main text, our model can readily be used to analyze various other statistics about a simulated epidemic: the total number and peak of hospitalization (see S1 Fig), total deaths and recoveries, total people that isolated, tracing false positives (S2 Fig) and false negatives (S3 Fig), the total number of non-compliant nodes, the tracing *efforts* (see [7]), the incidence and growth rates registered over a variable window size etc.

Both S2 Fig and S3 Fig give an alternative view of the repercussions a country can face if contact tracing is too zealous or too slow. If excessively many people get incorrectly isolated (false positives), the resulting socio-economic burden may significantly disrupt a community. If, on the other hand, tracing is very inefficient, the infectious population (false negatives) will spread the disease uncontrollably, leading to many hospitalizations and deaths. The right balance needs to be struck between these two for a "test and trace" strategy to be deemed successful.

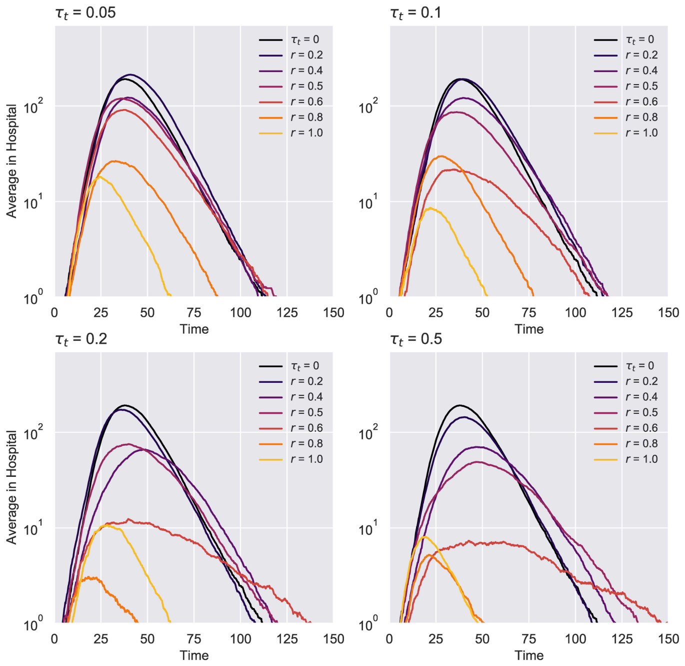

S1 Fig: Average number of hospitalizations per unit of time.
N=1000, with random graph topology and mean degree $K = 20$. τ_t fixed at 0.1.

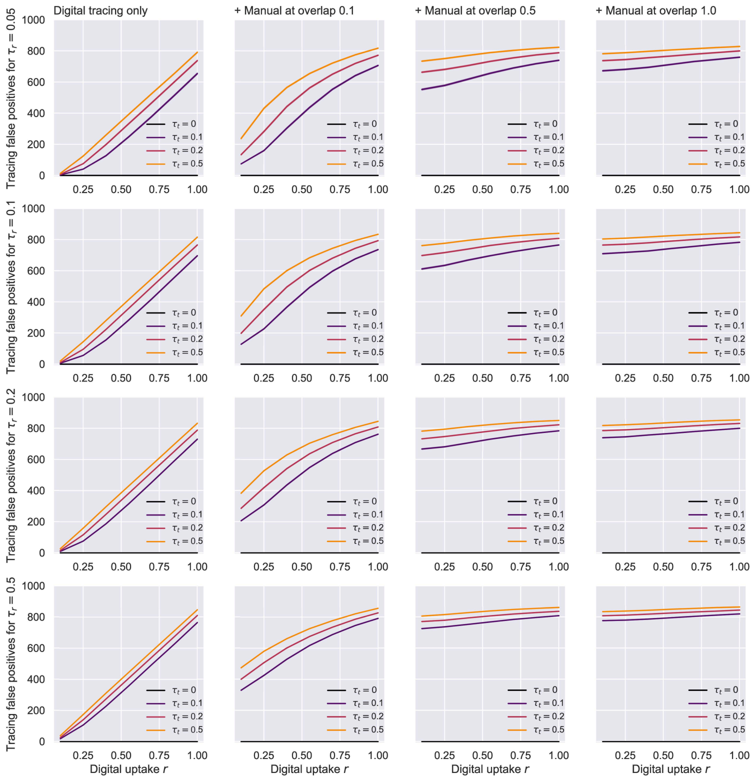

S2 Fig: Tracing false positives.

The amount of susceptibles being incorrectly traced and isolated. Results here correspond to the last experiment involving artificial networks, with $N = 1000$, a Holme-Kim graph topology and 10% initial infected.

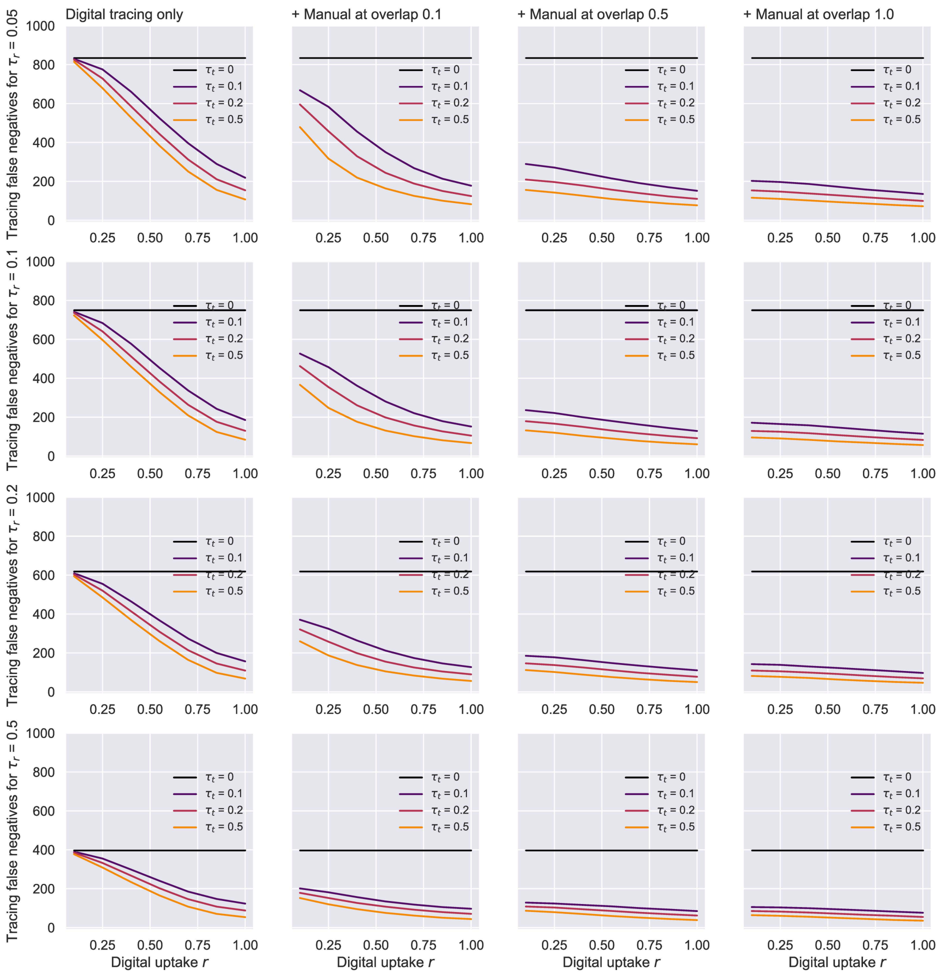

S3 Fig: Tracing false negatives.

The amount of *infectious* people not traced. These results correspond to the last experiment involving artificial networks, with $N = 1000$, a Holme-Kim graph topology and 10% initial infected.

Further analysis of noteworthy trends

As discussed in the main text, the values ascribed to overlap Γ or uptake r (depending on the type of tracing) dictate whether a contact tracing rate τ_t is actually effective. The trends imposed by these quantities on τ_t can be further scrutinized in S4 Fig and S5 Fig. At the 0.5 level, both are able to noticeably influence the infection curves obtained by $\tau_t \geq 0.04$. At the extreme points, the differences in peaks achieved by the same tracing rates become very large and apparent between one another.

When studying the combined effects of manual tracing at different Γ and digital tracing at various r on the effective reproduction number R, it is often useful to visualize the corresponding three-dimensional trends as a whole. To that end, we plot in S6 Fig the 3D surface of the aforementioned variables belonging to the last experiment.

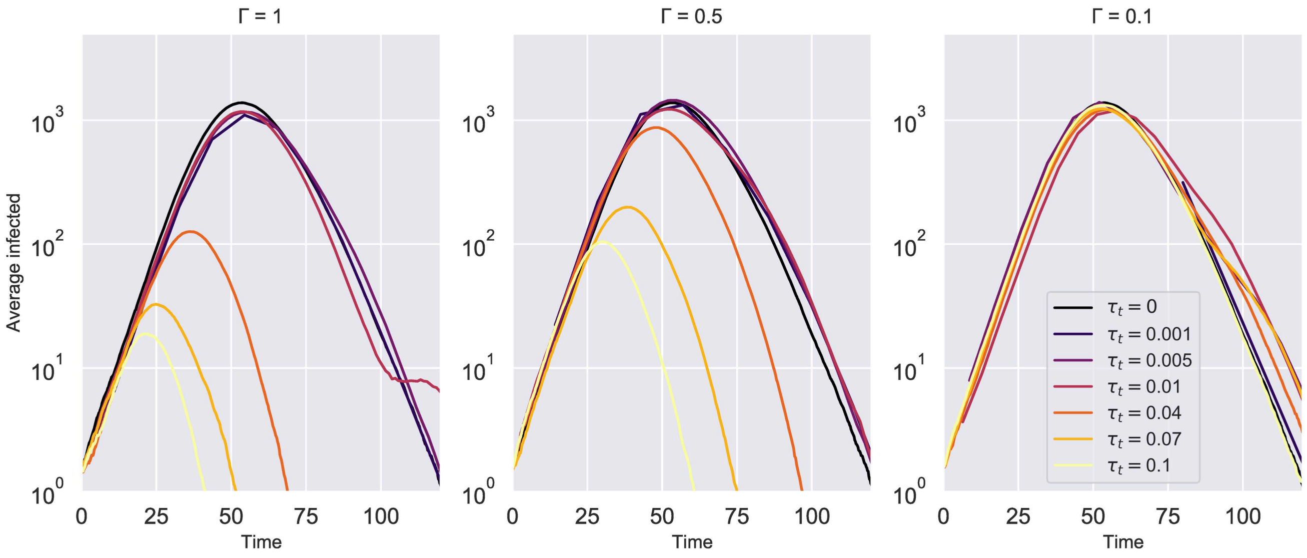

S4 Fig: Overlap influencing the efficacy of contact tracing rates.

N=10000, random graph topology with mean degree $K = 10$. τ_r fixed at 0.04.

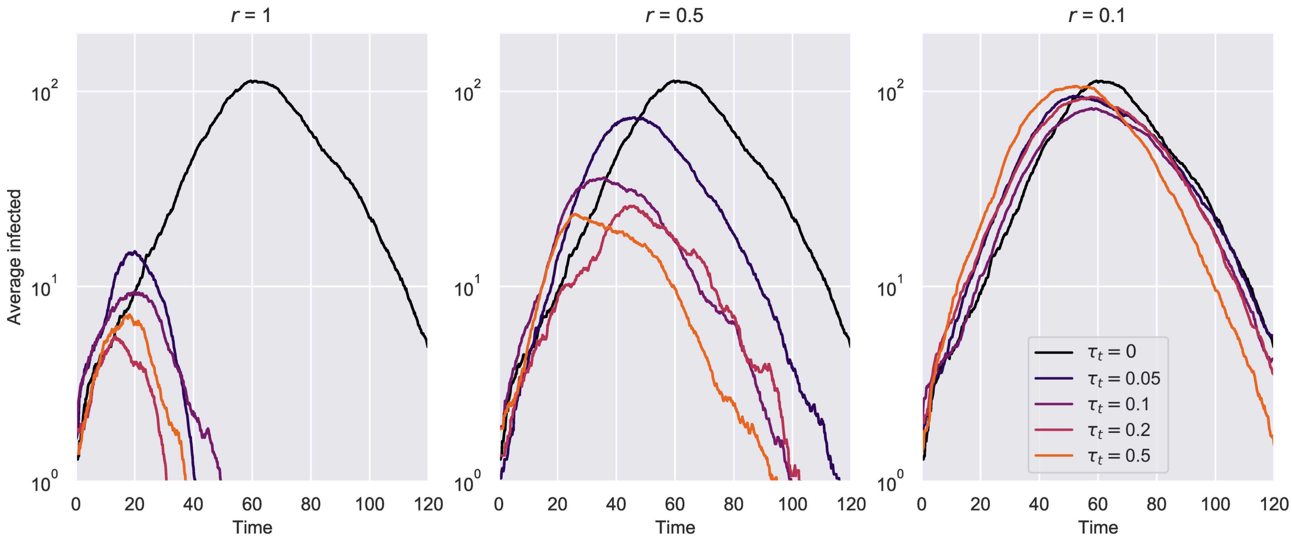

S5 Fig: Uptake influencing the efficacy of contact tracing rates.
N=1000, random graph topology with mean degree $K = 10$. τ_r fixed at 0.05.

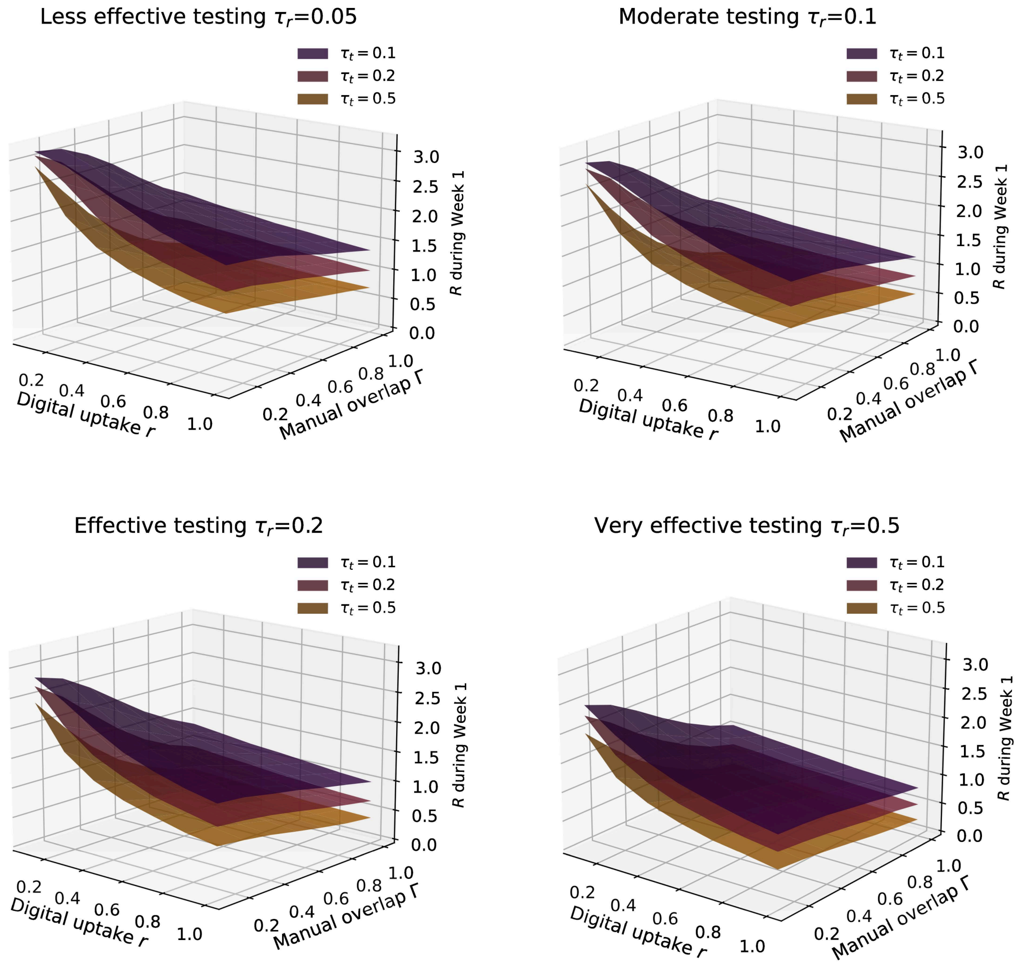

S6 Fig: Reproduction number *R* vs. uptake *r* vs. overlap Γ.
Results here correspond to the last experiment involving artificial networks, with $N = 1000$, a Holme-Kim graph topology and 10% initial infected.
