# Supplementary material for: Modelling digital and manual contact tracing for COVID-19. Are low uptakes and missed contacts deal-breakers?
Source: PLoS One. 2021 Nov 18;16(11):e0259969. doi: 10.1371/journal.pone.0259969 (PMC8601513; doi:10.1371/journal.pone.0259969)
Supplement: Attachment — Submitted filename: Response to Reviewers.pdf [file pone.0259969.s002.pdf]

Academic Editors
PLOS ONE
4 July 2021

Dear Dr. Kryven,

We thank yourself and the reviewers for all the insightful comments regarding our recent epidemiological work. The feedback we received was very detailed and it enabled us to significantly improve the manuscript in a relatively short timeframe. Consequently, we are very pleased to submit for your consideration our revised paper on “Modelling digital and manual contact tracing for COVID-19. Are low uptakes and missed contacts deal-breakers?”.

All points raised during the review process have been carefully scrutinized by our research team. Please find enclosed in the pages to follow our detailed responses to each particular question and suggestion posted in the original decision email. We would like to apologise in advance for the length of this letter, but we deemed it necessary to be as clear and explicit as possible in our replies, to avoid any further confusions about the discussed topics.

On behalf of my co-authors, I would like to end this cover letter by extending our gratitude for considering this resubmission. We appreciate all the time and effort you and the reviewers have put into this reviewing task!

Your sincerely,
Andrei Rusu (corresponding author)
rusu.c.andrei@gmail.com

Replies to editor

When submitting your revision, we need you to address these additional requirements.

1. Please ensure that your manuscript meets PLOS ONE's style requirements, including those for file naming.

Following the editor's comments, we amended the supporting information references to respect the format – i.e. S1 Fig instead of Fig S1. We also limited the use of italics to very important information only. Finally, we changed the formatting of the authors section to match the suggested styling.

Replies to reviewer #1

1. *The compartmental model used for describing the epidemic process is much more complicated than the classic ones. It is understandable, as the authors aim at describing realistic outbreaks of SARS-COV-2, in particular with the use of the Ip (pre-contagious), Ia (asymptomatic) and Is (symptomatic) compartments. However, the separation between H (hospitalised), R (recovered) and D is not necessarily useful, as D will only be a fraction from H set by lambda_H-D, and all remaining agents in the H compartment end up in the R one.*

Our goal from the very beginning was to make simulations that could capture a broad range of effects stemmed from the viral spread, including the evolution of hospitalizations and deaths. We also wanted to show that, unlike previous works, our contact tracing approach can be used in conjunction with virtually any complex compartmental formulation (many of which have been proposed for COVID-19 given the inefficacy of SIR [1]). That being said, this modeling decision is in line with many different works which explicitly separated these states [2, 3], including the study we used as reference when designing our proposed model [4]. It is true that, given the setup described in our paper, *D* are but a fraction from *H*. However, having the capability of directly capturing those metrics from different compartments is not only convenient, but also more realistic and useful when visualizing the specific effects of different measures (running our code allows for visual explorations of which node gets infected/hospitalized/dead and when). Having separate states with different transition times could also be exploited in the dynamical/temporal sense to infer noteworthy trends, although such an analysis is beyond the scope of the current paper. Lastly, this setup allows for a node's behavior within a simulation to change based on its state (for example, one may want to explore a scenario in which

hospitalized/recovered people can still ping their contacts through a tracing application, whereas a deceased node would be unable to aid the tracing process).

[1] Moein S, Nickaeen N, Roointan A, et al. Inefficiency of SIR models in forecasting COVID-19 epidemic: a case study of Isfahan. Scientific Reports. 2021;11(1):4725. doi:10.1038/s41598-021-84055-6
[2] Balabdaoui F, Mohr D. Age-stratified discrete compartment model of the COVID-19 epidemic with application to Switzerland. Scientific Reports. 2020;10(1):21306. doi:10.1038/s41598-020-77420-4
[3] Leontitsis A, Senok A, Alsheikh-Ali A, Al Nasser Y, Loney T, Alshamsi A. SEAHIR: A Specialized Compartmental Model for COVID-19. Int J Environ Res Public Health. 2021;18(5). doi:10.3390/ijerph18052667
[4] Di Domenico L, Pullano G, Sabbatini CE, Boëlle P-Y, Colizza V. Impact of lockdown on COVID-19 epidemic in Île-de-France and possible exit strategies. BMC Medicine. 2020;18(1):240. doi:10.1186/s12916-020-01698-4

2. *The mechanisms for generating the tracing networks are not described. How are the links chosen, when, and by which mechanism? Is the tracing connected to new infections or not? If not (as this is a mean-field approach), the model might have an intrinsic limitation due to the absence of correlation between the infection process and the network.*

We thank the reviewer for pointing out the generation mechanism was not intuitively explained. We have revised Section 2.2 of the paper to address this point. We now describe in more detail how the tracing networks are generated, based on the 2 inter-linked parameters: “degree of overlap” and “uptake”. On one hand, the degree of overlap gets converted to *Z_rem* via the specified formula, quantity which represents the average number of edges that get *randomly* removed from the infection network to render a tracing network (this effectively means *N * Z_rem / 2* edges get removed, and we have now made this explicit in the revision). On the other hand, the uptake dictates how many nodes should have all their edges removed (again at *random*). We average over many such tracing scenarios to reduce the potential noise induced by these random processes (hence why we mention in Section 3.1 that we simulate X different networks with Y random seeds). Consequently, the tracing networks are preset, so new infections do not *technically* influence their structure, but this should not pose a limitation because, in practice, we can assume this ‘static’ structure has been generated from aggregating all the *potentially-observed* dynamic links for any given node (i.e. a *random* process corresponding to tracers/apps monitoring the contacts neighborhood of any new *detected* infections), thus actually correlating the entire procedure to new infections. Given that presumption, the tracing propagation system becomes, in fact, *directly influenced* by any new *detected*

infection / isolated node, since the latter contributes to the additive probability of encouraging its neighborhood to self-isolate (hence the *multi-site* nature of our *mean-field* approach), while the neighborhood itself gets partially observed from the aforementioned pseudo-dynamic process.

That being said, removing the *random nature* of this operation altogether, and thus *actively* tying new infections to the tracing view substructure, is indeed an interesting avenue worth exploring (possibly via targeting the testing and the neighborhood exploration processes with reinforcement learning techniques, like [1] - a potential future direction for us as indicated in the revised Section 4), yet this may come at the cost of adding more bias: Currently, we assume contact tracers have a limited time to conduct their research and they do not favor any node when looking for contacts, whereas each individual's memory constitutes an unpredictable, seemingly-random factor to model. Hence, the simplifying presumption that contacts tend to be missed at random fits these assumptions and should be less biased. This very presumption enables us to view the precomputed static subnetwork as a dynamic aggregate of randomly-removed edges.

[1] Meirom EA, Maron H, Mannor S, Chechik G. How to Stop Epidemics: Controlling Graph Dynamics with Reinforcement Learning and Graph Neural Networks. arXiv:201005313 [cs]. Published online October 26, 2020. Accessed May 15, 2021. http://arxiv.org/abs/2010.05313

3. *On the same topic, at line 127 the authors say: "The tracing graphs are usually subsets of the first network." When is this not the case? From the model described in ref 21, I get that tracing might generate "false" contacts, but this is not said at any point in the manuscript.*

Thank you for pointing out this inconsistency. It is true that the referenced paper studies such a scenario in more detail. Our model is also capable of simulating "false" contacts, but we have decided not to study such a situation further in this paper since we deemed it less likely to happen in our global pandemic scenario, where contact tracers are generally very well trained and the public is much more aware of who they meet and for how long. We have modified Section 2.2 to better reflect this.

4. *The authors validate most of their findings using Erdös-Rényi networks, claiming that "it tends to offer acceptable approximations most of the time" (l146). I must argue that it does not, and in fact the authors themselves then use a scale-free/high clustering model in section 3.4. Why not directly use this one, which is indeed more realistic? They also could use empirical datasets, which are easily found in the community.*

We use ER networks as our baseline due to their wide adoption in the epidemic literature (including the studies we used as reference points for the spreading model [1, 2]), as well as their well-behaved epidemic dynamics in the limit, caused by the presence of epidemic thresholds (from [1], the study which introduced the *SIRT* model we extended, "the epidemic dynamics in SW networks remains qualitatively similar to random graphs, since they possess a well-defined epidemic threshold"). In addition to this, both real networks and ER graphs (under the studied K values) generally exhibit a single giant component, sparse densities, small average shortest paths [3,4]. Even though ER networks make a few unrealistic assumptions about the degree distribution (nodes with too many or too few connections are heavily restricted) and the independence of interactions in closed communities (where some inherent dependence certainly exists), they are still a good tool for modelling infections in randomly mixed populations [5] (e.g. in stores, public transportation etc. [6]). The first few experiments we conducted on the basis of ERs played a pivotal role in exemplifying the overall dynamics induced by different variables in our system, which we preferred to observe (for ourselves) and evoke (to the readers) in the most generalized framework, just as the predecessor papers did in their initial simulations. Indeed, the reviewer was kind to notice that we made a note of the unsuitability such a model may exhibit for social network tasks, hence why we focused the main experiments in the study on a more realistic network model, for which we actually derived our final conclusions and plotted the most representative figures on the efficacy of various contact tracing strategies w.r.t. the peak suppression and the reproduction number. We agree with the reviewer that our original statement about ERs was overly optimistic, and hence we have modified the section accordingly. We have also added Section 3.5 to the paper which details experiments we ran over a real dynamic social network.

[1] Tsimring LS, Huerta R. Modeling of contact tracing in social networks. Physica A: Statistical Mechanics and its Applications. 2003;325(1):33-39. doi:10.1016/S0378-4371(03)00180-8

[2] Farrahi K, Emonet R, Cebrian M. Epidemic Contact Tracing via Communication Traces. Lambiotte R, ed. PLoS ONE. 2014;9(5):e95133. doi:10.1371/journal.pone.0095133

[3] D. Du, "Social Network Analysis: Lecture 3-Network Characteristics," Sep. 28, 2016. [Online]. Available: http://www2.unb.ca/~ddu/6634/Lecture_notes/Lec3_network_statistics_handout.pdf

[4] A. L. Barabási, Network Science Random Networks. 2015. [Online]. Available: https://barabasi.com/f/624.pdf

[5] M. J. Keeling and K. T. D. Eames, "Networks and epidemic models," Journal of The Royal Society Interface, vol. 2, no. 4, pp. 295–307, Sep. 2005, doi: 10.1098/rsif.2005.0051.

[6] M. Abueg et al., “Modeling the combined effect of digital exposure notification and non-pharmaceutical interventions on the COVID-19 epidemic in Washington state,” in medRxiv, Sep. 2020, p. 2020.08.29.20184135. doi: 10.1101/2020.08.29.20184135.

5. *Another problem is the temporality of the spreading. Line 155, the authors state: "The time intervals between two state changes of the same kind are assumed to form an exponential distribution". This is very unrealistic. As shown in a wide range of empirical studies, human interactions exhibit bursty behaviour, with distributions of temporal properties having typical heavy-tails. It has been further shown that such properties condition strongly the spread of an epidemic (see for example Lambiotte et al EPJB 86 (2013) 320).*

We thank the reviewer for opening up this important discussion on the temporality involved in our simulations, which has prompted us to briefly explain our rationale in Section 2.3 of the revision. Our choice here was motivated by the exponential’s wide adoption in previous epidemiological works relying on SIR [1, 2, 4], as well as evidence of exponentially-distributed numbers of interactions over time observed in 2 reference studies for our work, which analyzed human behavior using tracking devices [2, 3]. We agree with the reviewer that our assumption can be too restrictive, especially surrounding infection times, and more flexible gamma (e.g. Erlang) or Weibull distributions represented via temporal sub-compartments could provide more realistic measurements. However, standard exponentials have been shown to offer acceptable approximations in several cases (e.g. when the mean infection duration is smaller [5], or the mean-generation time is correctly estimated [6]). As to other time intervals involved in the model (e.g. the infectious period), an older study showed that major epidemic persistence and dynamic problems could be introduced in SIR/SEIR simulations when using more realistic gamma distributions, amounting to worse results overall [7]. That is not to say using such distributions should be discouraged in SIR, but a very thoughtful consideration should be put into making this temporality choice nonetheless, carefully weighing the benefits against the potential limitations. We excluded this analysis for the purpose of the current paper, but it is definitely an interesting avenue worth exploring in subsequent studies.

[1] J. Ma, “Estimating epidemic exponential growth rate and basic reproduction number,” Infectious Disease Modelling, vol. 5, pp. 129–141, Jan. 2020, doi: 10.1016/j.idm.2019.12.009.

[2] K. Farrahi, R. Emonet, and M. Cebrian, “Predicting a Community’s Flu Dynamics with Mobile Phone Data,” Vancouver, Canada, Mar. 2015. doi: 10.1145/2675133.2675237.

[3] J. Stehlé et al., “Simulation of an SEIR infectious disease model on the dynamic contact network of conference attendees,” BMC Medicine, vol. 9, no. 1, p. 87, Jul. 2011, doi: 10.1186/1741-7015-9-87.

[4] M. Kröger and R. Schlickeiser, ‘Analytical solution of the SIR-model for the temporal evolution of epidemics. Part A: time-independent reproduction factor’, J. Phys. A: Math. Theor., vol. 53, no. 50, p. 505601, Nov. 2020, doi: 10.1088/1751-8121/abc65d.

[5] E. Vergu, H. Busson, and P. Ezanno, “Impact of the Infection Period Distribution on the Epidemic Spread in a Metapopulation Model,” PLoS One, vol. 5, no. 2, Feb. 2010, doi: 10.1371/journal.pone.0009371.

[6] O. Krylova and D. J. D. Earn, “Effects of the infectious period distribution on predicted transitions in childhood disease dynamics,” J R Soc Interface, vol. 10, no. 84, Jul. 2013, doi: 10.1098/rsif.2013.0098.

[7] A. L. Lloyd, ‘Realistic Distributions of Infectious Periods in Epidemic Models: Changing Patterns of Persistence and Dynamics’, Theoretical Population Biology, vol. 60, no. 1, pp. 59–71, Aug. 2001, doi: 10.1006/tpbi.2001.1525.

6. *I suggest the author define the measures they used to quantify the effect of tracing in the text. In particular, what is "peak suppression"? I would recommend a clearer measure, such as a normalised peak reduction (N_(no tracing) - N_tracing)/(N_(no tracing)). Same approach could be used for time of peak.*

Thank you for this insightful comment. We have addressed this point by adding a clear mathematical explanation of the peak suppression in the newly added Section 2.4. We would also like to mention that the original purpose of our peak suppression plots was to emphasize the benefit of contact tracing in absolute terms (scrutinized in direct reference to the total population size, which remains fixed at either 1000 or 10000 throughout the study, making it easy for the reader to visualize the full effect). While we agree with the reviewer that normalised charts would offer a valuable alternative view of our simulation data, an unnormalized reporting directly referencing the achieved peak suppression better fits with our initial plan.

7. *l203 : The authors select "good" values for tau_r and tau_t, but it seems to me that there is a correlation between the threshold values of tau_r and tau_t. It would be interesting to see heatmaps combining both dependencies.*

Thank you very much for this suggestion. We have now added heatmaps of the 2 parameters for different degrees of overlap to the revised Section 3.4.

8. *I have a question about the results presented in Fig 5, top right quadrant. It seems that the larger the population, the smaller the maximum infected fraction of the population, with a limit going to 0 as N increases. This would indicate that the model does not generate large cascades of infections, while spreading processes —being analog to percolation— should always reach a non-zero fraction of the population when the infection parameter is above the epidemic threshold. If accurate, doesn't it indicate an intrinsic unrealistic property of the model? It could also be that the parameters chosen for the study in section 3.1 lead to a spreading under the epidemic threshold, but in that case the analysis of the variation induced by population size is irrelevant.*

As illustrated by the unnormalized values in the left quadrant, the average of the peak of infection is monotonic on the studied range, and hence, for larger populations, the disease did manage to percolate at an increased rate (in absolute terms) as expected, making the variation analysis hold (at least on this very range). In spite of this, as indicated in the right quadrant and correctly pointed out by the reviewer, the peak clearly decreases in relative importance w.r.t. the total population (and the above-named monotony is not enough to ensure a non-zero *relative* average in the limit). Even so, the purpose of this experiment was not to study the dynamics in the limit, but rather to check if the variability across runs scales with the number of nodes (as initially suspected), and to settle on a sensible range of values for the population size (which would not be plagued by large variances, at least relative to that size). Indeed, as the reviewer correctly intuited, the limiting behavior of the percolation process is the result of analyzing a scenario in which both tau_t and tau_r are *marginally larger* than the infection rate beta (0.1 against 0.0791). Should the uptake have been 100% and considering that both the infection and contact tracing “spread” in a similar fashion, the simulation would have been deterministically contained (and below the epidemic threshold with a greater probability). However, given that the uptake in this experiment is set to 50%, this is now not always the case, as some instances do get quickly contained, while others continue to percolate for longer periods, depending on the network subsetting seed. It is also worth noting here that Section 3.1 reports on the variance of *peaks,* rather than overall

epidemic sizes. While tracing can indeed be effective at “flattening the curve”, this does not guarantee the final size actually follows the same trend (or has the same decaying shape in the limit).

On a side note, our choice of studying \tau_t=\tau_r=0.1 here stemmed from empirical observations of this particular assignment resulting in an “average” efficacy w.r.t. preventing full-blown outbreaks, across many different parameter configurations (possibly due to the aforementioned marginal difference compared to the transmission rate). As reported in the paper, this assignment belongs to the higher-mid range of our initial setups, but also lies within the lower-mid range in the main experiments.

9. *The study would be much stronger if the model was explored and validated for a larger range of spreading parameters. Only two values for p_a and one for p_h are considered. I understand that the authors have used estimated values from the empirical studies, but since the model for interactions is not realistic, it might be that these values are not suitable to reproduce the desired spreading properties (see my previous comment). Similarly, only two values for K are considered. I understand that the model is complex and the parameter space is huge, but the current results merely rely on eye-balling "relevant" values from a minimal set of tries. Deriving quantitative, meaningful results from such an evaluation of "proper" values for the parameters seems too optimistic.*

As the reviewer kindly noted, the parameter space is indeed very large, and it is very difficult to find the most realistic values here. We concentrated our experiments on the most agreed-upon values, which were previously derived by trusted institutions and used to inform real governmental policies (by the CDC [1], by the The French National Institute of Health and Medical Research [2]). Our compartmental model was deliberately designed to be “in line” with [2], and therefore we were very composed with parameter changes (in fact, modifications were conducted only based on estimates from other trusted sources). This makes our results meaningful for the scenario in question. That being said, we consider our main contribution to be the modelling technique itself (aspect reflected in our decision to submit to this particular journal), and hence the range of the parameters was constrained to the most sensible values for presentation. A wider range of tau_r and tau_t values was, however, studied in our experiments, some significantly below and above the epidemic threshold, with the final ranges reported in the final piece being indicative of all the observed trends. We agree with the reviewer that a more thorough analysis of the parameters should be carried away in future work, especially in relation with K, and this has been marked accordingly in Section 4 (Conclusions and future work).

[1] M. A. Johansson et al., “SARS-CoV-2 Transmission From People Without COVID-19 Symptoms,” JAMA Netw Open, vol. 4, no. 1, p. e2035057, Jan. 2021, doi: 10.1001/jamanetworkopen.2020.35057.

[2] L. Di Domenico, G. Pullano, C. E. Sabbatini, P.-Y. Boëlle, and V. Colizza, “Impact of lockdown on COVID-19 epidemic in Île-de-France and possible exit strategies,” BMC Medicine, vol. 18, no. 1, p. 240, Jul. 2020, doi: 10.1186/s12916-020-01698-4.

10. *Manual tracing and digital tracing seem to be a sort of redundant system, to increase the global tracing efficiency. Shouldn't the use of both be equivalent to having a single, higher value of either digital tracing or manual tracing?*

The two tracing systems operate on different assumptions (please refer back to Section 3.4). Namely, both conduct tracing over different networks (a sensible assumption since not all interviewees will have a COVID tracing application to notify neighbors, and there may be discrepancies between what each service “sees”). In manual tracing, the overlap controls missed contacts, while in digital tracing both an overlap (inadequate use) and an uptake rate can influence the topology (the latter does not make sense in the manual tracing case). Finally, there is an inherent delay of tracing a contact in the interview-based approach (controlled by differences in \tau_t rates; assumed to be equal to two days in our study, but with direct support in the code for varying that delay).

11. *I would appreciate that the authors discuss two features from Fig 7:*

Thank you for the questions below. First, we would like to note that we have changed Fig 7 in the revision to match the given caption, since in the initial submission we have imported by mistake an older version of that figure (with less scenarios depicted). In the new figure, the 4 quadrants visible in the originally-submitted picture are all placed on the right column. We have also removed the case with overlap=0 since this theoretically corresponds to the same scenario as \tau_t=0. That being said, the questions below still hold since this figure update is based on the same data and scenarios.

11.1. *How can contact tracing lead to situations in which the outbreak lasts longer?*

First of all, we must distinguish all cases from the first row, which we regard as giving mostly noisy results of the contact tracing process due to the ineffective value of \tau_t. In some of the more-meaningful configurations, very small values of the overlap (<.33) still manage to

fall within the “noise region” because \tau_t is not large enough to make their effect distinguishable from the noise induced by the inherent stochasticity of the simulations. Such a poor representation of the infection network can also lead to situations in which only incorrect regions of the graph get isolated (i.e. many false positives), which in turn can cause the epidemics to gain momentum at later stages (e.g. an obvious example is overlap=0.11 in the third quadrant). A brief explanation has been added to the revision of Section 3.2.

11.2. *What generates the bimodality of some curves?*

The effect of multiple peaks has also been noted for the SIR formulation in [1]: “with larger tracing effort, the epidemic is reduced significantly rapidly, leaving a great deal of the population susceptible for a second peak of infections[...] This is due to the contact tracing becoming so effective that the number of cases drops rapidly, resulting in tracing becoming less effective. Note, this effect is much more attenuated in the dual network case” (the “dual network case” refers to a scenario with a single contact tracing network in which the overlap is very small: 0.08). Indeed, in our case this bimodality is a rarer phenomenon which only affects a few of the simulations that exhibit large values of the overlap.

[1] Farrahi K, Emonet R, Cebrian M. Epidemic Contact Tracing via Communication Traces. Lambiotte R, ed. PLoS ONE. 2014;9(5):e95133. doi:10.1371/journal.pone.0095133

12. *Furthermore, I have some minor issues with the manuscript:*
- I would advise for replacing ref 21 with "R. Huerta, L. Tsimring, Phys. Rev. E 66 (2002) 056 115". I am not an expert on multi-site mean-field models, and I found that the latter reference contains a much clearer description of the approach than the one given by the authors.
- l196 : please move the reference to Fig 7 at the relevant location ; the current one is a sentence about Fig 6

We thank the reviewer for spotting these minor issues. Indeed, the initial paper by Huerta and Tsimring includes more details on multi-site mean-field models, but the latter features more experiments and contains further conclusions derived from them. This is why we decided to keep both references in the revision. As to Fig 7, as mentioned above, the caption was in fact correct but we imported an older version of that figure onto the system. This has now been amended.

Replies to reviewer #2

1. *The conclusions of the simulation study are weakened by the fact that they rely solely on simulated contact networks which lack many realistic features of real world contact patterns. I think the model in itself and the present study merit publication on their own, but it would be a big plus if the authors could motivate that the networks' parameters are realistic and that the missing features do not change the conclusions, and/or perform simulations on empirical contact networks.*

We thank the reviewer for this important suggestion, and we agree that the study would benefit from showcasing the impact of contact tracing on real social networks. To that end, we have added Section 3.5 (with background explanations added to Section 2.3) where we explore a real data-driven scenario.

2. *The model has a lot of different variables and parameters and it is difficult to keep track of them all. It would be very helpful to list all of them in a table, similar to Table 1. Namely: \Gamma, K, r, t_rem. It might also be helpful to list the different compartments (states) of the model.*

Thank you for this suggestion. A table detailing the network parameters used in our simulations has been added to the revision. A diagram of the compartments together with the available transitions has been supplied in Fig 1, with the caption detailing their meaning.

3. *In the same manner, it would make the manuscript easier to read if the names, or short definitions, were given for each of the parameters when their ranges considered are listed in each subsection of the Results, e.g., on lines 187 and 188 recall that \Gamma is the degree of overlap, \tau_t the contact tracing rate, etc.*

Thank you for this thoughtful suggestion. The names of the different parameters have now been made apparent at the beginning of each section.

4. *Add values of all relevant parameters to figure captions. This will make the figures easier to read.*

We thank the reviewer for this suggestion. The values of the relevant parameters have now been added to the figure captions in the revised study.

5. *It is unintuitive to use S_i for general states and S for the susceptible state. I suggest the authors use another symbol to denote a general state, e.g. X_i.*

We have amended the table according to this suggestion, using X to refer to different states.

6. *The fact that the relative error is ~3% for networks of size N=1000 and ~1% for N=10000 is corresponds to a relative error of 1/\sqrt{N}, consistent with central limit type arguments for a well mixed population. The result may not hold however in a structured population or near a critical point and thus cannot necessarily be extrapolated to general conditions.*

We thank the reviewer for this insightful comment. We have now made a note of the limitations our variance analysis has.

7. *The authors refer to some parameters of their model as "hyperparameters". This term is generally reserved for parameters of an inference procedure, not a model, e.g., of the learning procedure in machine learning or of prior distributions in Bayesian inference.*

The term hyperparameters was chosen to distinguish network parameters from compartmental model parameters. We have now modified the nomenclature accordingly to avoid any confusion.

8. *The authors compare the uptake rate in the simulations to the digital tracing adoption rates reported for different countries. However, the adoption rates cited are typically calculated as numbers of download relative to the population, which is not the same as the fraction of population using the app correctly.*

The adoption rates cited are estimated from application downloads, which can actually represent significant overestimations of the true uptake (this is now apparent in Section 3.4 of the revised text). As to modelling the fraction of the population using the app correctly per se, our simulations should technically rely on the degree of overlap \Gamma instead of the uptake r. The former, however, was kept to 1 in the digital tracing network, as was correctly observed by the reviewer in the next question.

8. *In Section 3.4, if I understood correctly, the overlap between edges in the digital contact tracing network and the true contact network is assumed to be perfect, while the uptake in the manual tracing network is assumed to be 100%. Both of these assumptions seem overly optimistic to me, e.g., the manual contact tracing system will saturate at a given prevalence as was seen in most western countries, while digital contacts are only proxies for real (possibly disease transmitting) contacts.*

Indeed, we are only varying the overlap for manual tracing and the uptake for digital tracing, but the two parameters are interlinked within a single network (varying one can directly impact the other). That being said, the "uptake" in manual tracing does not have a clear real-life correspondent, unless we are referring to memory-impaired individuals who are unable to identify any of their contacts (but for the purpose of our study we assumed such special circumstances can be covered by the randomness of link removal attributed to diversifying the degree of overlap). In contrast, varying the "overlap" in digital tracing corresponds to inadequate usage patterns of the application, and was made entirely possible in our codebase. However, showcasing the effects of sampling the entire parameter grid becomes combinatorically harder, so we decided to illustrate only a targeted subset of the scenarios our model is actually capable of running.

8. *Please corroborate the claim (on lines 144-146) that the Erdos-Renyi graph model tends to offer acceptable approximations most of the time.*

From [1], the study which introduced the SIRT model we extended, "the epidemic dynamics in SW networks remains qualitatively similar to random graphs, since they possess a well-defined epidemic threshold". In addition to this, both real networks and ER graphs (under the studied K values) generally exhibit a single giant component, sparse densities, small average shortest paths [3,4]. Even though ER networks make a few unrealistic assumptions about the degree distribution (nodes with too many or too few connections are heavily restricted) and the independence of interactions in closed communities (where some inherent dependence must actually exist), they are still a good tool for modelling infections in randomly mixed populations [5] (e.g. in stores, public transportation etc. [6]). That being said, we do agree our original statement is overly optimistic, and therefore we amended our formulation in the revision.

[1] Tsimring LS, Huerta R. Modeling of contact tracing in social networks. Physica A: Statistical Mechanics and its Applications. 2003;325(1):33-39. doi:10.1016/S0378-4371(03)00180-8

[2] Farrahi K, Emonet R, Cebrian M. Epidemic Contact Tracing via Communication Traces. Lambiotte R, ed. PLoS ONE. 2014;9(5):e95133. doi:10.1371/journal.pone.0095133

[3] D. Du, "Social Network Analysis: Lecture 3-Network Characteristics," Sep. 28, 2016. [Online]. Available: http://www2.unb.ca/~ddu/6634/Lecture_notes/Lec3_network_statistics_handout.pdf

[4] A.-L. Barabási, Network Science Random Networks. 2015. [Online]. Available: https://barabasi.com/f/624.pdf

[5] M. J. Keeling and K. T. D. Eames, "Networks and epidemic models," Journal of The Royal Society Interface, vol. 2, no. 4, pp. 295–307, Sep. 2005, doi: 10.1098/rsif.2005.0051.

[6] M. Abueg et al., "Modeling the combined effect of digital exposure notification and non-pharmaceutical interventions on the COVID-19 epidemic in Washington state," in medRxiv, Sep. 2020, p. 2020.08.29.20184135. doi: 10.1101/2020.08.29.20184135.

8. *It seems like an error that symbols in Table 1 are in boldface.*

This was done on purpose to highlight the afferent symbols, but in the new version of the table we removed this styling.

9. *The authors may want to discuss the implications of the crossover effects seen in Fig. 10, i.e., the final outbreak size can be larger for higher uptake.*

A discussion on the rare "crossover effects" has been added to the revised manuscript in Section 3.3.

10. *Can the authors comment on how realistic the required values of \Gamma and \tau_t for combined tracing to be effective alone are? Have the values of these parameters been estimated empirically?*

The parameter values we indicated as being "effective" have been estimated empirically through inspecting the simulation results. Unfortunately, it is a rather challenging task to find real-world estimates of either of these two parameters. This is why we conduct an extensive study of the trends emerged from running simulations over a large range of values. The UK government SAGE has recommended that at least 80% of close contacts be reached for the system to be deemed effective – overlap=0.8:

https://www.health.org.uk/news-and-comment/charts-and-infographics/nhs-test-and-trace-performance-tracker. This percentage is highly influenced, however, by the recalled contacts (only 74% provided at least one close contact in the last month) and the possibility to reach those contacts (84% identified and asked to isolate in the last month). This would bring the actual overlap to a value significantly lower than 0.8 (the quoted value of 0.5 being reasonable to assume as possible to achieve).

11. *In Fig. 5 add description of what symbols and boxes and whiskers represent.*
Line 74: Change "SIR" to "The SIR process"
Line 80: add abbreviation "(Inserm)"
Line 121: "onto" → "on"
Lines 140-141: should "scales with" be replaced by "is proportional to"?
Line 205: Specify what is meant by "meaningful results".
Line 212: "N" → "small N".
Line 302: "encapsulated" → "model's"

Thank you for these helpful suggestions. We have accommodated these in the revision.
