# Supplementary material for: Modelling digital and manual contact tracing for COVID-19. Are low uptakes and missed contacts deal-breakers?
Source: PLoS One. 2021 Nov 18;16(11):e0259969. doi: 10.1371/journal.pone.0259969 (PMC8601513; doi:10.1371/journal.pone.0259969)
Supplement: Attachment — Submitted filename: ResponsetoReviewers-Stage2.pdf [file pone.0259969.s003.pdf]

Academic Editors
PLOS ONE
26 October 2021

Dear Dr. Kryven,

We thank yourself and the reviewers for all the insightful comments regarding our recent epidemiological work. The feedback we received, both now and during the first reviewing stage, was very detailed and it enabled us to significantly improve the manuscript in a particularly short timeframe. Consequently, we are very pleased to submit for your consideration our revised paper on "Modelling digital and manual contact tracing for COVID-19. Are low uptakes and missed contacts deal-breakers?".

All points raised during the review process have now been carefully scrutinized by our research team. Please find enclosed in the pages to follow our detailed responses to each of the suggestions arisen during the second reviewing phase. Please also note that the senior co-author has been moved to the last position in the authors list, as per her request.

On behalf of my co-authors, I would like to end this letter by extending our gratitude for considering this resubmission. We appreciate all the time and effort you and the reviewers have put into the whole reviewing process!

Your sincerely,
Andrei Rusu (corresponding author)
rusu.c.andrei@gmail.com

Replies to editor

1. Please review your reference list to ensure that it is complete and correct. If you have cited papers that have been retracted, please include the rationale for doing so in the manuscript text, or remove these references and replace them with relevant current references. Any changes to the reference list should be mentioned in the rebuttal letter that accompanies your revised manuscript. If you need to cite a retracted article, indicate the article's retracted status in the References list and also include a citation and full reference for the retraction notice.

We reverified our References list to ensure no retracted papers got cited. At the same time, we added a few more references to support our discussion on waiting times and network structures, in light of the suggestions made by the reviewers. In addition, we supplemented the information provided for references 5, 6, 10, 16 (previously 15), 17 (previously 16) with links to the corresponding cited web resources. Finally, we modified references 18 (previously 17) and 45 (previously 65) to reflect these papers' recent publication.

2. While revising your submission, please upload your figure files to the Preflight Analysis and Conversion Engine (PACE) digital diagnostic tool, https://pacev2.apexcovantage.com/. PACE helps ensure that figures meet PLOS requirements. To use PACE, you must first register as a user. Registration is free. Then, login and navigate to the UPLOAD tab, where you will find detailed instructions on how to use the tool. If you encounter any issues or have any questions when using PACE, please email PLOS at figures@plos.org. Please note that Supporting Information files do not need this step.

All our figures have been verified using the PACE system, and no issues were reported, except for Fig8.eps in which the tool detected bitmap images with resolution < 300 DPI. We have inspected the apparently-problematic figure but found no issues with its rendering. Please also note that most of our figures were regenerated using a different colormap in light of the comments made by Reviewer #3, and adequate margins have now been added.

Replies to Reviewer #2

1. The authors have added a discussion of the modeling assumption that state transitions take place with constant rates (exponentially distributed waiting times). Which is great since this common assumption is often not satisfied in empirical data. However, they discuss only the case where waiting time distributions are less skewed than exponentials. Though they may also be more skewed, notably due to intercontact times and edge weights following heavy-tailed distributions as is often the case in physical proximity networks often (see e.g. Starnini et al. "Modeling human dynamics of face-to-face interaction networks" PRL 2013).

We thank the reviewer for pointing out that intercontact times may often be gracefully modelled via heavier-tailed distributions, such as power laws. We have now added a comment on this aspect in the manuscript. The decision we took for our contact tracing model was based on the findings of 2 cohort studies that reported exponential decays in the number of interaction events over time, while also having in mind the fact that this is a very common assumption in the epidemiological literature. The work of Starnini et al. [1] found power laws to be excellent approximators for the contact duration, but a power law fit to waiting times was found to be moderately less optimal, as the authors remark themselves. We would also like to emphasize that the figures presented in the aforementioned study utilize a log-log scale, which may sometimes hide exponential trends. In fact, the authors of this paper do not provide the parameters for the fitted power law to the intercontact times (although they do so for contact duration), so it is harder to judge the extent of the inferred exponent. At the same time, the fitted curve exhibits a concave curvature in the log-log plot, which may actually correspond to a roughly-exponential trend if translated to a linear scale. Sadly, no comparison with an exponential fit is provided in this text either. We acknowledge, however, that some interaction datasets do feature more skewed waiting times distributions than Gamma, and hence we believe that exploring the impact of utilizing longer-tailed distributions is an interesting avenue worth exploring in future endeavors.

[1] Starnini et al. "Modeling human dynamics of face-to-face interaction networks" PRL 2013

2. One has to read Eqs. 1 and 2 to understand how Γ and r are defined, while the definitions of Z_{rem} and N_{utn} are clear from the text. I suggest moving the first part of Eqs. 1 and 2 (before the $\Rightarrow$ sign) up to where Γ and r are introduced. The second parts of Eqs. 1 and 2 may be removed as they are obtained by a simple arithmetic inversion of the first parts, or the authors may keep them at their current place.

Thank you for suggesting this easier-to-comprehend rephrasing and reordering of Section 2.2 and its equations. The manuscript has been amended to include in-line equations for all the network parameters involved, while full equations for N_{utn} and N_{ute} have been provided below.

3. Change: ‘contacts network’ to ‘contact network’ on page 6 (no line numbering) and on page 7, line 137. Change: ‘newly isolated’ to ‘isolated’ on page 6.
Change: ‘testing regimes’ to ‘testing regime’ in Fig. 7 caption.
Change: ‘tau_r’ to ‘τ_r’ in line 252.
Change ‘First aspect’ to ‘The first aspect’ in line 304.
Change: ‘experiments’ to ‘simulations’ in line 371.

Thank you for signaling these difficult-to-spot phrasing issues and mistakes. These suggestions have now been accommodated in the final manuscript.

Replies to Reviewer #3

1. First, as other reviewers mentioned, the network structure and temporal dynamics are somewhat overlooked in the paper. Although I would not argue that the authors should perform extra simulations, I think it is important to acknowledge it more thoroughly in the discussion. It has been recognized that super-spreading is a rather universal characteristic of many epidemics [1] and COVID-19 is argued to be driven primarily by such super-spreading events. Furthermore, recent studies (e.g., [2]) have shown that when spreading is driven by such super-spreading events, the details of contact tracing implementation may matter a lot. In this context, I believe that the paper should expand the discussion to provide a better context to the readers.

[1] Lloyd-Smith, J. O., Schreiber, S. J., Kopp, P. E. & Getz, W. M. Superspreading and the effect of individual variation on disease emergence. Nature 438, 355–359 (2005).
[2] Kojaku, S., Hébert-Dufresne, L., Mones, E., Lehmann, S., & Ahn, Y. Y. (2021). The effectiveness of backward contact tracing in networks. Nature Physics, 17(5), 652-658.

We thank the reviewer for pointing out that our discussion on superspreading, a very important mechanism underpinning the spread of COVID-19, was minimal in the previous submission. As such, we have now included a larger paragraph on superspreading, preferential attachment and small-world networks (see Section 2.3). In the same section, we have also commented on why we believe our contact tracing modelling technique remains suitable in the face of challenges like superspreading in scale-free networks or large clustering coefficients in small-world graphs, taking into consideration the findings of Kojaku et al. [1] and Tsimring and Huerta [2], respectively, as well as the similarities between our modelling technique and theirs.

[1] Kojaku, S., Hébert-Dufresne, L., Mones, E., Lehmann, S., & Ahn, Y. Y. (2021). The effectiveness of backward contact tracing in networks. Nature Physics, 17(5), 652-658.

[2] Tsimring LS, Huerta R. Modeling of contact tracing in social networks. Physica A: Statistical Mechanics and its Applications. 2003;325(1):33-39. doi:10.1016/S0378-4371(03)00180-8

2. Second, I think the plots can be improved a lot by carefully choosing colors and by limiting the number of lines/objects that each figure shows. Many figures have numerous (~10) lines with random colors associated with each line, making them very difficult to parse. I believe that most of the figures will not lose much information by reducing the number of lines to ~5. In addition, as each of these lines show a range of parameter values (i.e., they can be ordered), a linear colormap (e.g., sampling colors across the "viridis" colormap) would make them much easier to read. Also, the heatmap figure uses green-to-red colormap, which can be understood by a significant fraction of population who has colorblindness. Furthermore, the colormap used introduces an arbitrary cut-off point (between 750 and 1000) that introduces an artifact. Again, I believe that a linear, perceptually uniform colormap should be used here. Although this is probably not "critical" regarding PLOS ONE's publication criteria, I believe that this simple improvement in the figures will make the paper much more accessible.

Indeed, the original color schemes used for our plots were sometimes counterintuitive and probably difficult to comprehend for people with color blindness. To fix this issue, we have regenerated all our charts, including the heatmaps, using the linear colormap ‘inferno’. What is more, we have also reduced the number of lines in all the overlap/uptake figures to a maximum of 7 different rates, thus making them more comprehendible.
